# Inflammatory Responses Induced by the Rupture of Intracranial Aneurysms Are Modulated by miRNAs

**DOI:** 10.1007/s12035-019-01789-1

**Published:** 2019-10-25

**Authors:** Michal Korostynski, Rafal Morga, Marcin Piechota, Dzesika Hoinkis, Slawomir Golda, Tomasz Dziedzic, Agnieszka Slowik, Marek Moskala, Joanna Pera

**Affiliations:** 1 Department of Molecular Neuropharmacology, Institute of Pharmacology, Polish Academy of Sciences, ul. Smetna 12, 31-343, Krakow, Poland; 2 Department of Neurosurgery and Neurotraumatology, Faculty of Medicine, Jagiellonian University Medical College, ul. Botaniczna 3, 31-503, Krakow, Poland; 3 Intelliseq sp. z o.o, ul. Chabrowa 12/3, 31-335, Krakow, Poland; 4 Department of Neurology, Faculty of Medicine, Jagiellonian University Medical College, ul. Botaniczna 3, 31-503, Krakow, Poland

**Keywords:** Subarachnoid hemorrhage, Intracranial aneurysm, miRNA, Peripheral blood, Cytokines

## Abstract

Influence of an intracranial aneurysm (IA) rupture on the expression of miRNAs and the potential significance of the resulting changes remains poorly understood. We aimed to characterize the response to the IA rupture through the analysis of miRNAs in peripheral blood cells. Expression of small RNAs was investigated using deep transcriptome sequencing in patients in the acute phase of an IA rupture (first 72 h), in the chronic phase (3–15 months), and controls. A functional analysis and the potential interactions between miRNAs and target genes were investigated. We also measured the levels of proteins that were influenced by regulated miRNAs. We found that 106 mature miRNAs and 90 miRNA precursors were differentially expressed among the groups. The regulated miRNAs were involved in a variety of pathways, and the top pathway involved cytokine-cytokine receptor interactions. The identified miRNAs targeted the inflammatory factors *HMGB1* and *FASLG*. Changes in their expression were detected at the mRNA and protein levels. IA rupture strongly influences the transcription profiles in peripheral blood cells. The regulated miRNAs were involved in the control of immune cell homeostasis. In summary, these results may aid in the elucidation of the molecular mechanisms that orchestrate the inflammatory response to IA rupture.

**Electronic supplementary material:**

The online version of this article (10.1007/s12035-019-01789-1) contains supplementary material, which is available to authorized users.

## Introduction

The rupture of an intracranial aneurysm (IA) resulting in a subarachnoid hemorrhage (SAH) results in many systemic effects and strongly influences the functioning of the immune system. Alterations in inflammation-related protein levels in peripheral blood and in the number and activity of immune cells have been reported [1–3]. These changes are associated with the systemic consequences of IA rupture, including inflammatory responses and immunodepression, which might be critical for patient outcomes. The prognosis of SAH remains poor for most patients, who suffer from high mortality rates and long-term neuropsychological complications and decreased quality of life [4]. The molecular mechanisms underlying the systemic response to IA rupture are still insufficiently understood. Their elucidation would be useful for the improvement of the treatment of SAH patients.

There is a growing body of evidence that indicates that noncoding RNAs (ncRNAs) play a crucial role in many pathological conditions. Among the ncRNAs, microRNAs (miRNAs) are the best-described class of posttranscriptional regulators. These small molecules (18–25 nucleotides in length) result in the posttranscriptional silencing of targeted protein-encoding genes. Moreover, a single miRNA controls the expression of multiple genes, and a single gene can be regulated by various miRNAs. The role of miRNAs in pathologies related to cerebral vessels seems to be beyond dispute [5–7].

Previously, we demonstrated that IA rupture induces a systemic response associated with changes in the mRNA profiles in peripheral blood cells and the composition of mononuclear cells, in an inhibition of lymphocyte responses and a concomitant enhancement of monocyte activity [8]. Here, we sought to investigate the effects of IA rupture on the expression of small ncRNAs in peripheral blood cells using deep transcriptome sequencing. Moreover, we analyzed the levels of selected inflammatory molecules, for which expression might be regulated by miRNAs that were identified has having differential expression in aneurysmal SAH. To assess the time-related changes, patients in the acute and chronic phases of SAH were included in the study.

## Methods

### Patients

Patients with ruptured IAs were prospectively recruited from patients at the Department of Neurology or Neurosurgery and Neurotraumatology, University Hospital, Krakow, as previously described [8]. Briefly, two independent SAH patient groups were analyzed: acute (first 72 h after IA rupture) and chronic (3–15 months after SAH). Control subjects (C) were recruited from outpatients clinic patients who suffered from headaches. However, they were not sampled during the acute pain attack and they were not on a long-term specific anti-pain medication. All subjects were free of any immune system-affecting disorders (including autoimmunity and malignancies) and immunomodulating drugs (including steroids and immunosuppressants). Demographic and risk factor data were collected. All subjects were Caucasian. Written informed consent was obtained from all participants (or the guardians of participants) before their inclusion in the study. The local ethics committee approved the study.

The differences between the groups were analyzed using χ^2^ and Fisher exact tests or the Mann-Whitney *U* test where appropriate. A *p* value <0.05 was considered statistically significant.

### Blood Collection and RNA Extraction

Venous whole blood was collected before neurosurgical intervention in PAXgene Blood RNA Tubes (PreAnalytiX, GmbH, Switzerland). The tubes were frozen and stored at −70 °C until further processing. Total RNA was purified from blood samples using a PAXgene Blood RNA Kit (PreAnalytiX) according to the manufacturer’s protocol and was treated with DNase. The RNA concentrations were measured using a NanoDrop ND-1000 Spectrophotometer (NanoDrop Technologies, Montchanin, DE), and the RNA quality was determined by chip-based capillary electrophoresis utilizing the Agilent RNA 6000 Nano Kit and an Agilent Bioanalyzer 2100 (Agilent, Palo Alto, CA) according to the manufacturer’s protocols.

### Small RNA Sequencing

Small RNA (sRNA) library preparation and sequencing were performed with Illumina sequencing technology (Illumina, San Diego, CA). The sRNA library was generated with the TruSeq Small RNA Library Kit. Briefly, 3′ and 5′ adapters were ligated to 1 μg of total RNA with T4 RNA ligase. Then, reverse transcription was performed with Illumina sRNA RT-Primer, and the cDNA was amplified by PCR (11 cycles) using the Illumina sRNA primer set. The amplified total cDNA library was purified and size-selected (insert size 22–30 bp) in a 6% Novex TBE gel. The transcriptome libraries were sequenced on a HiSeq 2500™ (Illumina) with the following parameters: SE50 (single end) and 10 M clean reads; this provided a minimum of 500 Mb of raw data per sample. The RNA-seq data were submitted to the NCBI Sequence Read Archive (SRA): SRP150595.

### Next-Generation Sequencing (NGS) Data Analysis

The sequence read quality was evaluated using the FastQC (0.11.5) quality filter module. The raw reads were mapped to the reference human genome hg38 using BWA (version 0.7.17). The expression levels were quantified using IntersectBed (v2.28.0) and GTF with data from the miRbase database (version 20) and the DASHR database of human small noncoding RNAs in human tissues and cell types (v2.0). sRNA transcripts with counts per million (CPM) values above 2 in at least 10 samples were considered detectable and were used for further analysis. The statistical significance was analyzed using the edgeR package (v.3.6.8). The false discovery rate (FDR) was estimated using the Benjamini-Hochberg method. The paired samples t-test was used to compare the means between the groups of samples (C vs RAA, C vs RAC, RAA vs RAC). All statistical analyses were performed using R software v3.4.3. Hierarchical clustering was performed using the measure of 1-correlation distance metric and centroid distance linkage methods. Cluster visualization was performed using dChip software v. 2010.01 (DOI: 10.1186/1471-2105-9-231).

### Functional Classification of Differentially Expressed miRNAs

To identify the set of active biological pathways associated with the miRNAs and their target genes, we applied stringent statistical methods. The miRNA Enrichment Analysis and Annotation Tool (miEAA) was used to identify overrepresented ontological groups within the set of regulated miRNAs and to group them according to functional classifications [9]. The list of miRNAs identified by sRNA sequencing was used as the input for the analysis. The statistical significance analysis of miRNA enrichment was performed using the ORA algorithm as implemented in the miEAA online resources. Overrepresented categories were defined as having at least 10 miRNAs and an FDR adjusted *p* < 0.05.

### miRNA Target Prediction

The prediction of molecular targets was performed to examine miRNA-mediated regulation in a pathway-specific manner. Genes included in the top pathway, which was cytokine-cytokine receptor interactions (hsa04060), and the related GO category cytokine activity (GO:0005125) were considered to be involved in immune responses and analyzed for the presence of conserved sites that matched the seed regions of the regulated miRNAs. A list of biologically predicted targets of differentially expressed miRNAs found in our study was compiled using miRBase. Experimentally validated targets were considered along with predicted targets (Target score > 60). To determine the possible miRNA-mRNA interactions, the list of gene targets was compared with the list of differentially expressed miRNA genes. The results of the gene expression profiling were obtained from RNAseq of the total transcriptome from the same patients [8].

### Enzyme-Linked Immunosorbent Assay

The blood samples were collected at the same time as those obtained for transcriptomics and were centrifuged within 1 h of collection. Plasma was collected and stored at −80 °C until use. Based on the miRNA profiling, the following cytokines were selected: soluble Fas (sFas), soluble Fas ligand (sFasL), and high mobility group box 1 protein (HMGB1). Their concentrations in plasma were determined using commercially available ELISA kits; for sFas and sFasL, a Quantikine kit (R&D Systems Inc., Minneapolis, MN) was used, and for HMGB1 a kit from IBL International (Hamburg, Germany) was used according to the manufacturer’s protocols. The intra- and inter-assay coefficients were below 7%. All measurements were performed in duplicate. The differences between groups were examined using the Mann-Whitney *U* test, and *p* < 0.05 was considered statistically significant.

## Results

### Alterations in sRNA Abundance Levels in Response to IA Rupture

The study included 19 patients in the acute phase of IA rupture (RAA, first 72 h), 20 patients in the chronic phase of SAH (RAC, 3–15 months), and 20 controls. Their clinical characteristics were previously described [8] and are briefly summarized in Table 1. Ruptured IA were located in the anterior circulation in 17 RAA patients and 16 RAC patients. During hospitalization, 4 patients in the RAA group developed infections (2 – pneumonia, 1 – urinary tract infection, 1 – sepsis), and 2 patients died.

**Table 1:** Clinical characteristics of the patients.

	RAA (n = 19)	RAC (*n* = 20)	C (n = 20)
Median age, years (IQR)	54 (48–62)	50 (41–56)	55 (50–60)
Female, %	73.7	90.0	55.0*
Hypertension, %	57.9	55.0	50.0
Smoking, %	31.6	40.0	30.0
Excessive drinking, %	0	5.0	10.0
Diabetes mellitus, %	5.3	5.0	10.0
Hyperlipidemia, %	5.3	0	15.0
Admission Hunt-Hess score (IQR)	2 (1–3)		
Admission WFNS score (IQR)	1 (1–3)		
Admission GCS score (IQR)	15 (13–15)		

RAA = acute phase of intracranial aneurysm (IA) rupture; RAC = chronic phase of IA rupture; C = control subjects; IQR = interquartile range; GCS = Glasgow Coma Scale; WFNS = World Federation of Neurological Surgeons

*p < 0.05 RAC vs C

We used NGS to comprehensively examine the expression levels of sRNA derived from the peripheral blood. The normalized miRNA abundance levels (CPM) were measured for the sRNA transcripts annotated in the GRCm38.p13 genome release. A total of 65,156 RNAs were analyzed, and the expression signal was detected for 17,930 transcripts. For 1766 sRNA genes, we detected abundance levels at the threshold defined by CPM >2 (in at least 10 samples). When comparing the RAA and RAC patients and the controls, we found 106 mature miRNAs and 90 miRNA precursors that were differentially expressed between the groups (FDR <10% in edgeR). In general, the profiles of the alterations are similar for the precursors and the respective mature miRNAs. The pair-wise comparisons and comparison of fold-change of differences between the groups (C vs RAA, C vs RAC, RAA vs RAC) indicated that majority of changes when miRNA abundance levels increased were detected in the acute phase. In the chronic phase of IA rupture we revealed relatively more downregulated miRNAs comparing to the acute phase (Supplementary Table S1). We focused further analysis on the miRNA expression profiles and the putative target genes.

### Expression Profiles of miRNA Alterations in Peripheral Blood Cells from Patients after IA Rupture

**Fig. 1:**
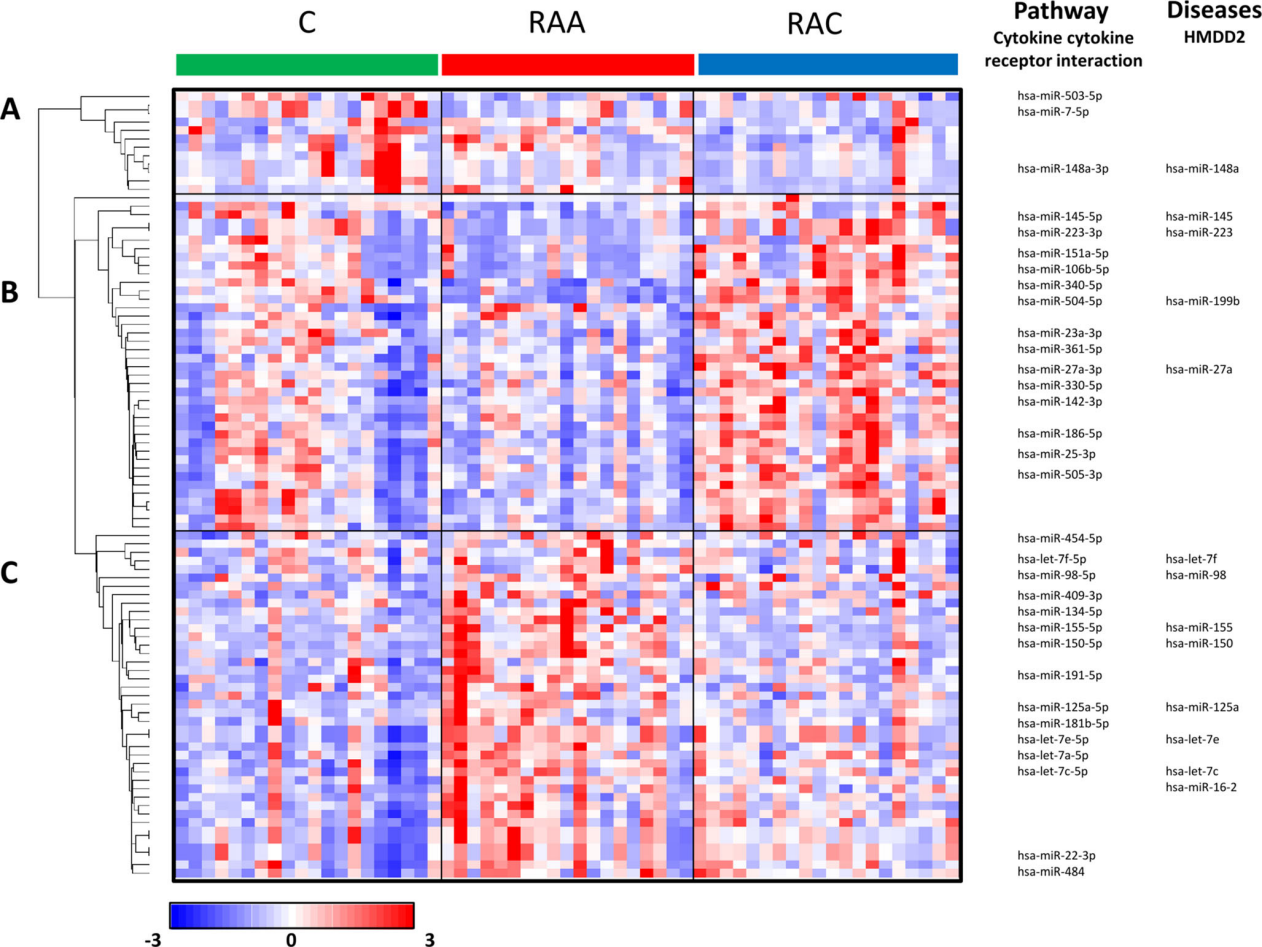
Expression profiles of miRNAs in the peripheral blood cells of patients after IA rupture. The differences in expression in samples from subjects in the acute phase of IA rupture (RAA), the chronic phase of IA rupture (RAC) and healthy controls (C) were analyzed by using small RNAseq. The results are shown as a heatmap and include miRNAs with genome-wide significance in terms of the clinical status factor (10% FDR). The colored rectangles represent the transcript abundances of 93 miRNA measured in the samples described above (the list is included in Supplementary Table S1). The expression profiles of the miRNAs are hierarchically clustered using a 1-correlation distance metric and the centroid linkage of nodes. Major miRNA transcription patterns are arbitrarily described as ‘A’, ‘B’, and ‘C’. The intensity of the color is proportional to the standardized values (between - 3 and 3) of each RNAseq measurement, as indicated on the bar below the heatmap image. Blue-to-red shades indicate increased relative expression; blue shades indicate reduced expression; white indicates median expression. The regulated miRNAs linked to various molecular pathways and the disease etiology are listed on the right. The presented miRNAs are linked to the top enriched pathway (hsa04060 - cytokine-cytokine receptor interaction) and/or associated with disease pathophysiology (based on the HMDD database) [10]. The disease-related example miRNAs are connected to at least four diseases in the top seven (Supplementary Table S2). The threshold level for statistical significance for enrichment was set at an FDR adjusted *p* < 0.05

miRNAs are functionally well defined members of the small ncRNA family. The presented analyses were based on the functional annotations available for miRNAs. The list of regulated sRNAs contains 93 unique miRNAs (Supplementary Table S1). Hierarchical clustering revealed three major patterns in the miRNAs (A-C) altered in response to IA rupture (Fig. 1).

The two main profiles of the alterations contain miRNAs that are downregulated (pattern B with 42 miRNAs) and upregulated (pattern C with 39 miRNAs) in the acute phase of IA rupture. Both of these patterns return to normal levels (based on the controls) in most patients in the chronic phase of IA rupture. The additional small cluster profile consists of miRNAs (pattern A with 11 miRNAs) that are downregulated in the chronic phase of IA rupture.

Also, we tested sample classification using the identified signature of the regulated miRNAs. However, the distinction between chronic phase of IA rupture and controls was limited.

Since the female patients were overrepresented in the RAC group when compared to controls, we evaluated the potential effect of this difference on expression of miRNAs. We identified 24 miRNAs that show sex-dependent (FDR 10%) expression profile in our dataset. One of the differentially expressed miRNAs (miR-1307-5p) was also found on the list of IA-rupture regulated miRNA (FDR 10%). However, the effect of clinical status (RAA, RAC, C) was still significant when sex factor was taken into account. Thus, we considered that the difference in proportion of males and females between the experimental groups did not have a major effect on the profile of transcriptional alterations induced in response to IA rupture.

### Functional Annotation of the Regulated miRNAs in Peripheral Blood Cells

To identify the functional associations of the miRNAs with altered expression in response to IA rupture, we used enrichment analysis. To characterize the transcriptional representation of biological processes, a list of 93 miRNAs was analyzed by using miRWalk annotations. Among the regulated miRNAs, functional clusters connected to GO terms that included receptor binding, the regulation of ubiquitin protein ligase activity, and inflammatory responses (FDR corrected *p* < 0.01) were overrepresented. The analysis of the enriched signaling pathways revealed more specific functional connections. The analysis indicated that the miRNAs regulated after IA rupture (for example, miR-142-3p, let-7f-5p, and miR-98-5p) might be involved in cytokine-cytokine receptor interactions (hsa04060 pathway, FDR corrected p < 0.01) (Supplementary Table S2).

### Gene Target Prediction for the Identified miRNAs

**Fig. 2:**
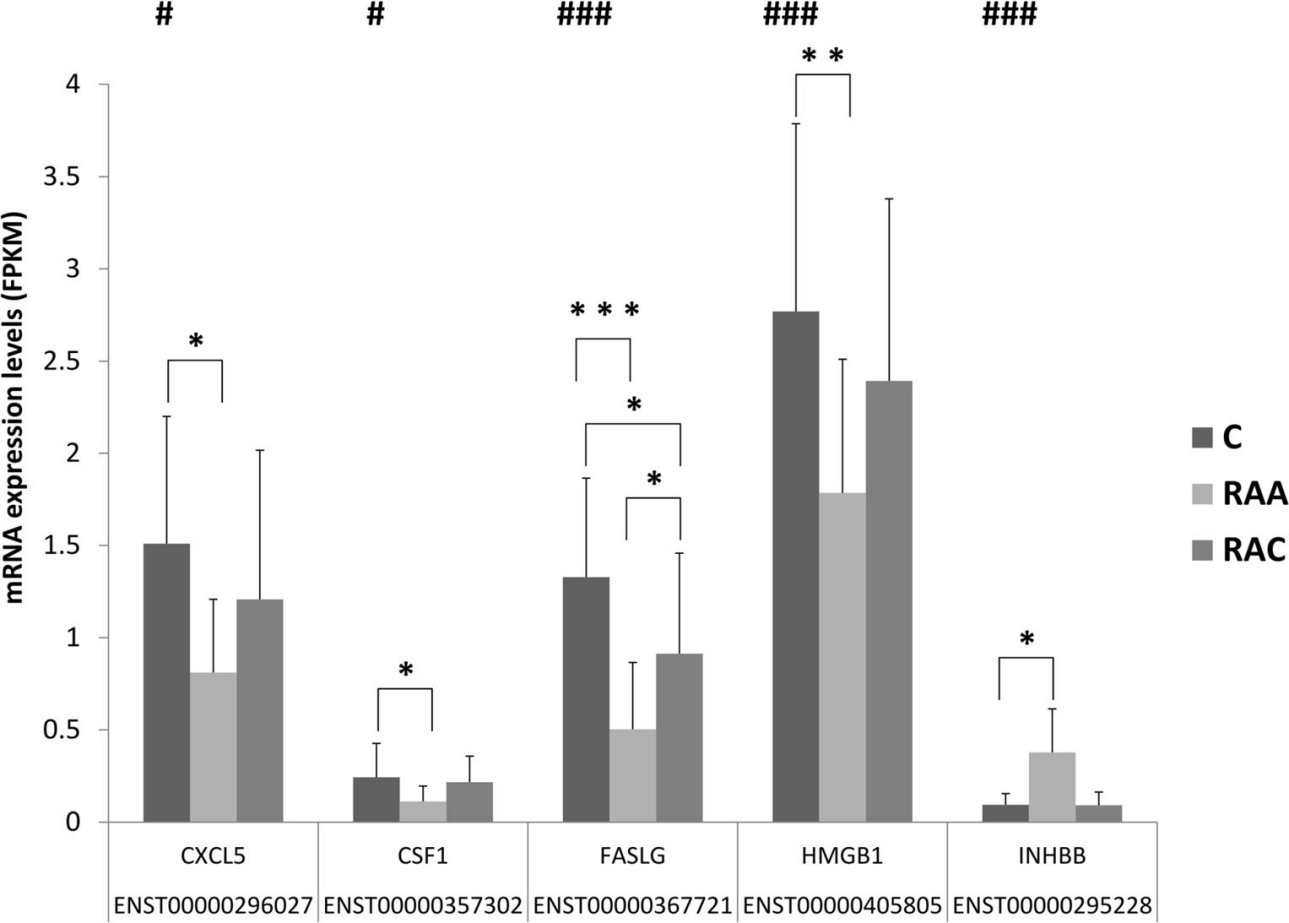
Alterations in the transcript expression of the predicted target genes for the identified miRNAs. The differences in gene expression between the samples from subjects in the acute phase of IA rupture (RAA), the chronic phase of IA rupture (RAC) and healthy controls (C) were analyzed by using total RNA sequencing [8]. The mRNA abundance levels of the target genes *HMGB1, CXCL5, CSF1* and *FASLG* were analyzed in peripheral blood cells from patients after IA rupture. The results are presented as relative expression levels (RPKM, reads per kb per million reads) with the standard deviation (*n* = 19–20). Significant primary effects based on analysis of variance with one-way ANOVA for the clinical status factor (FDR # < 10%, ## < 1%, ### < 0.1%) are indicated (Supplementary Table S3). Differences between the RAA, RAC and C groups were analyzed using Tukey’s multiple comparisons test (*p < 0.05, **p < 0.01, ***p < 0.001)

The analysis revealed that out of the 93 identified miRNAs, 34 target cytokines that are regulated in response to IA rupture. Twenty-three potential targets (with a Target score > 60) were identified from the list of 215 genes that were involved in cytokine activity (GO:0005125) and cytokine-cytokine receptor interactions (hsa04060). The list of targets includes *INHBB* (miR-22-3p, miR-148a-3p, miR-152-3p, and miR-22-3p), *CSF1* (miR-484), *CXCL5* (miR-25-3p), *HMGB1* (let-7a-3p, let-7f-1-3p, miR-142-3p, miR-3529-3p, and miR-505-3p), and *FASLG* (let-7a-5p, let-7c-5p, let-7e-5p, and miR-98-5p). The mRNA abundance levels of the target genes *HMGB1*, *CXCL5*, *CSF1* and *FASLG* were decreased in patients in the acute phase of IA rupture (RAA) in comparison to those in control patients. The expression level of *INHBB* transcript was increased in patients in that stage (Fig. 2).

### Regulation of the Targeted Inflammatory Factors

**Fig. 3:**
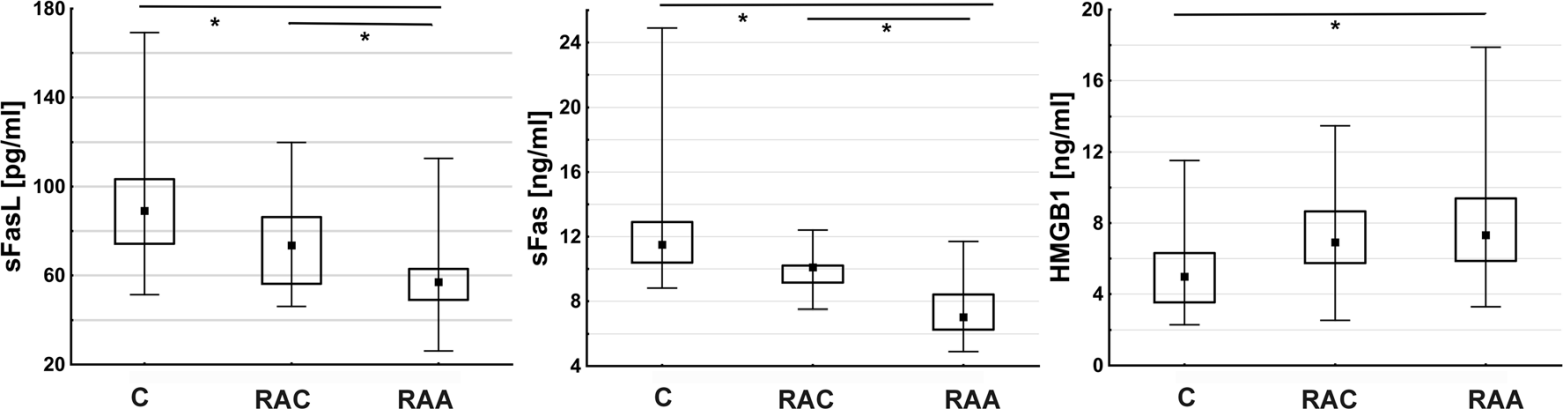
Plasma levels of sFasL, sFas, and HMGB1. *p < 0.05

For the analyses of the protein levels of identified potential miRNA targets, we chose HMGB1 and sFasL. In addition, we measured the sFas levels. Both sFas and sFasL showed significantly decreased levels in SAH patients in comparison to those in controls. In the acute phase, the levels were lower than in the chronic period after IA rupture. The sFas/sFasL ratio did not differ significantly between the analyzed groups. In contrast, the HMGB1 concentration was significantly higher in RAA patients than in control subjects, but no significant differences were observed between the RAA and RAC groups. A summary of these data is presented in Fig. 3.

### Search for Disease-Related miRNAs Altered in Response to IA Rupture

To find clinical points of reference for our results, we compared the lists of regulated miRNAs with previously reported associations with disease etiology. We used the Human MicroRNA Disease Database (HMDD), and we found that a significant number of the identified miRNAs are associated with clinical conditions related to immune system activity, inflammatory responses, and cell cycle regulation. A functional analysis revealed that IA rupture is associated with the dysregulation of miRNAs involved in the etiology of cancer, muscular disorders and heart failure (Supplementary Table S3). Analysis of the pathological conditions known to be linked to differentially expressed miRNAs (including hsa-let-7c, hsa-let-7e, hsa-miR-98 and hsa-miR-142) indicated that the common features of these conditions are the involvement of the immune system, inflammatory responses, and cell cycle regulation. The list of miRNAs associated with the diseases contains several that target genes that encode cytokine-related factors (let-7c, let-7e, miR-98 target *FASLG*, and miR-142 targets *HMGB1*).

## Discussion

Following IA rupture, we found significant changes in the expression levels of several subclasses of small ncRNAs. The most prominent effects of SAH were observed within the first 72 h. This period is critical in SAH and is closely related to early brain injury and neuroinflammation [11]. Here, we focused our analyses on miRNAs, which made up the largest subgroup of regulated sRNAs. This category of molecule is one of the best characterized. Although miRNAs constitute a small part of the human genome, they regulate most genes in a complex way. On the one hand, each miRNA can target hundreds of mRNAs; on the other hand, a single mRNA is often the target of multiple miRNAs [12]. Moreover, gene products may also influence miRNA expression. This kind of regulation is proposed for cytokines, during which miRNAs target cytokine mRNA and cytokine signaling in turn has an impact on miRNA expression [13].

Functional analyses of the regulated miRNAs revealed that the differentially expressed miRNAs are generally involved in processes related to inflammation, the immune system, the cell cycle, cell death, and apoptosis. Such miRNAs were at the top of lists of overrepresented regulated miRNAs complied by using different tools, including WikiPathways, Gene Ontology, and HDMM. This finding is consistent with the results of other studies that investigated SAH by analyzing changes in cellular activity, the transcriptome, or protein levels. These studies showed that IA rupture causes obvious dysregulation of both innate and adaptive immunity, which is important for disease severity and outcome. Lopes et al. investigated miRNA expression profiles in peripheral blood in 27 aneurysmal SAH patients and 6 controls between 7 and 10 days after IA rupture. Among the 8 miRNAs identified as differentially expressed between the SAH patients and controls, 3 miRNAs, let-7f-5p, hsa-miR-451a, and hsa-miR-941, were also found in our study and showed the similar expression changes [14]. In studies carried out in acute ischemic stroke (IS), differentially expressed miRNAs in blood cells were related to immune/inflammatory responses and the cell cycle. This finding is consistent with our knowledge about the effects of central nervous system injury on peripheral immune system function. Bam et al. investigated IS patients in the first 48 h and found several miRNAs to be dysregulated that are common in our RAA group, including hsa-miR-199b-3p, − 223, −30e, − 503, and − 550*. Among the top pathways enriched for regulated miRNAs were those related to cancer, hematological, and immunological diseases [15]. Additionally, Jickling et al. analyzed miRNA expression in acute IS patients and found 8 differentially expressed miRNAs that potentially regulate genes in pathways associated with inflammatory/immune responses [16]. The analysis of pathways involved in the response to IA rupture might be more important for a general understanding of the underlying biological mechanisms rather than the examination of single molecules. In particular, differences in study design and the methods used in different studies may make direct comparisons of the results at a single molecule level difficult.

Based on the results of the functional annotation, we focused our gene target prediction analysis on cytokines by taking advantage of the availability of mRNA expression data from the same study subjects. Only mRNAs that showed differential expression among the studied groups were investigated [8]. Then, we measured the plasma protein levels for selected molecules with known mRNA data. Therefore, we chose sFasL and HMGB1 and the soluble form the receptor for FasL, sFas.

The results of the protein measurements were surprising. The abundance levels of miRNAs that probably control the expression of *FASLG* and *HMGB1* were increased, and the mRNAs for these cytokines were downregulated in acute SAH patients. Thus, one can expect decreased levels of protein products. This was true for sFasL but not for HMGB1.

FasL is a member of the tumor necrosis factor protein family. It induces apoptosis in Fas-expressing cells and is an important death factor in the immune system [17]. It also plays a role in inflammation by inducing the production of proinflammatory cytokines and neutrophil infiltration [18]. FasL can be present in either a membrane-bound form (mFasL) on the cell surface or a soluble form (sFasL), which is generated by the cleavage of mFasL by matrix metalloproteinases [19]. These forms play distinct roles; mFasL mediates apoptosis, whereas sFasL has an anti-apoptotic function [18].

Fas is a receptor for FasL, and the Fas/FasL pathway is an important regulator of apoptosis within the immune system. For instance, activation-induced cell death in peripheral T cells is mediated through the interaction of Fas and FasL [20]. Likewise FasL there are different functional isoforms of Fas that are generated by alternative splicing and are expressed as soluble molecules (sFas) [21]. sFas prevents apoptosis by inhibiting the binding of Fas to FasL at the cell surface [22]. Thus, the circulating sFas/sFasL system is an important part of the apoptosis signaling pathway. Here, we found the significant downregulation of both sFasL and sFas proteins after IA rupture. The interpretation of these results is unclear since we did not measure the expression of the membrane-bound forms of these proteins. Thus, an unambiguous determination as to whether this represents pro- or antiapoptotic activity is impossible. However, our results from the flow cytometry analyses showed a significant decrease in CD3+ T cell counts (both CD4+ and CD8+) in the acute SAH period in the same patients [8]. To the best of our knowledge, there are no reports of the systematic study of the sFas/sFasL system in SAH patients. Studies on IS provided different data. Mahovic et al. found that sFas levels were higher in serum and cerebrospinal fluid (CSF) in acute IS patients than in controls [23]. In contrast, Tarkowski et al. repoerted decreased levels of sFas in CSF [24]. In FasL mutant (*gld*) mice subjected to focal brain ischemia, the percentage of CD4+ T cells in peripheral blood was decreased when compared to that in wild-type animals [25]. In a population-based Japanese study, higher serum sFas levels were associated with higher mortality in SAH [26].

In contrast, HMGB1 protein levels in plasma in the RAA group were increased, whereas *HMGB1* mRNA was downregulated in comparison with that in controls.

HMGB1 is a nonhistone DNA binding protein that is released following tissue injury from necrotic cells and serves as a proinflammatory cytokine [27]. It is also recognized as a damage-associated molecular pattern molecule (DAMP).

The results of studies on IS suggest the important role of HMGB1 in stroke-induced immunosuppression, which is similar to a phenomenon observed in SAH [28–31]. The levels of this protein were increased in IS patients and correlated with the number of circulating leukocytes, stroke severity, and inflammatory markers [32]. A recent meta-analysis of 28 studies comprising almost 2700 IS patients found significantly increased circulating HMGB1 blood levels that were correlated with stroke severity and infarct volume [33]. DAMPs, including HMGB1, can also initiate the inflammatory response after IA rupture. In many studies, an increase in HMGB1 levels in CSF SAH patients was found and was shown to be positively correlated with worse outcomes [34, 35]. Higher plasma levels of HMGB1 in aneurysmal SAH were associated with worse clinical status and poor prognosis [36].

In the present study, the expression of HMGB1 at the mRNA and protein levels was discordant.

One of the possible explanations could be the different sources of particular molecules. RNAs were isolated from peripheral blood cells, whereas protein was measured in plasma. Circulating HMGB1 can be released from various cells, including activated platelets [31, 37]. The activation of platelets is a known phenomenon in SAH and is associated with early brain injury after SAH [3, 38]. Thus, one can speculate that in the acute period after IA rupture, there is a tendency to decrease the production of HMGB1 in peripheral leukocytes; however, due to its release from other sources, including activated platelets, the plasma levels continue to rise.

Our study has some potential drawbacks. One is the relatively limited sample size and the lack of validation of the obtained results in another group of patients. However, in most studies on miRNA expression, the numbers of included subjects are similar. Although control group was recruited among patients suffered from headaches, they were not sampled during the acute episode of pain. Moreover, they were not on a specific anti-pain medication. Thus, we assumed that obtained results can be generalized and the detected differences between studied groups are related to the IA rupture. The results of the present study highlight the significance of immune/inflammatory responses in IA rupture and are consistent with previous reports from our group and others. In addition, we cannot sharply delineate which specific miRNA or miRNA cluster plays a key role and which signaling pathway is the most important during systemic reactions to SAH. This study was designed to analyze changes in global expression profiles to obtain more general insights into the peripheral processes underlying the response to SAH. However, we did not include in this study patients with unruptured IAs. Thus, some of the transcriptomic changes may be related to the presence of IA itself. We also analyzed the alterations in expression in peripheral leukocytes only. Thus, we cannot assess the effects of IA rupture on the transcriptome in other cell types that may also play important roles in the acute SAH phase. However, considering that vessel wall cellular components, including endothelium and vascular smooth muscle cells, might be involved, human studies using that approach are likely to be extremely difficult to conduct, if at all possible.

In summary, the results of this study demonstrated that IA rupture influences the expression of miRNAs in peripheral blood cells. The most highly regulated pathways are related to inflammation and the immune response, especially those related to cytokine processes. However, further studies are warranted to clarify the roles of specific miRNAs in SAH and to translate them into clinical applications.

## Electronic supplementary material

ESM 1 (DOCX 13 kb)

ESM 2 (XLSX 69 kb)

ESM 3 (XLSX 54 kb)

ESM 4 (XLSX 26 kb)
