# Supplementary material for: Inflammatory Responses Induced by the Rupture of Intracranial Aneurysms Are Modulated by miRNAs
Source: Mol Neurobiol. 2019 Oct 25;57(2):988–96. doi: 10.1007/s12035-019-01789-1 (PMC7031224; doi:10.1007/s12035-019-01789-1)
Supplement: Supplementary file 1 — (DOCX 13 kb) [file 12035_2019_1789_MOESM1_ESM.docx]

**SUPPLEMENTARY MATERIAL**

**to “Inflammatory responses induced by the rupture of intracranial aneurysms are modulated by miRNAs”**

**SUPPLEMENTARY TABLES – DESCRIPTION**

Supplementary Tables S1-S3 are available in uploaded Excel Files.

**Supplementary Table S1**

**Summary of the results of the miRNA expression profiling of peripheral blood cells from patients after IA rupture**

The differences in expression in samples from subjects in the acute phase of IA rupture (RAA, n=19), the chronic phase of IA rupture (RAC, n=20) and control subjects (C, n=0) were analyzed by using small RNAseq. The table presents a list of the 106 mature miRNAs and 90 miRNA precursors with significant differences in terms of RNA abundance levels between the experimental groups. The regulated miRNAs are listed with their identifiers, chromosome location, mean abundance level in each group, fold-change compared to the control, and the p-value obtained from edgeR and FDR.

**Supplementary Table S2**

**The results of functional enrichment analysis performed with the MiEAA tool for the list of miRNAs significantly altered in response to IA rupture**

The table consists of enriched pathways, diseases or GO terms (presented in three consecutive sheets), the number of miRNAs associated with each category (expected and observed), the levels of significance in terms of overrepresentation presented as FDR-corrected p-values (p<0.05), and the lists of miRNAs/precursors.

**Supplementary Table S3**

**Summary of the results of the miRNA target prediction**

The analysis was performed for a list of 215 genes from the cytokine activity GO category (GO:0005125). The table consists of the target genes and the corresponding targeting miRNAs with appropriate annotations and the abundance levels in the RAA, RAC and C experimental groups. Only miRNAs that were significantly altered in response to IA rupture were analyzed (FDR<10%). The analyzed mRNA transcripts were differentially expressed in response to IA rupture (ANOVA, FDR<10%). The results of the gene expression profiling were obtained from RNAseq of the total transcriptome from the same patients.
